# How Patients Shape Healthcare: Then, Now and What's to Come?

**DOI:** 10.1111/jan.70502

**Published:** 2026-02-03

**Authors:** G. Tobiano

**Affiliations:** ^1^ NHMRC Centre of Research Excellence in Wiser Wound Care Griffith University Southport Queensland Australia; ^2^ Gold Coast Hospital & Health Service, Gold Coast University Hospital Southport Queensland Australia; ^3^ School of Nursing and Midwifery Griffith University Southport Queensland Australia

## 1 Introduction

Historically, healthcare has been paternalistic; patients were expected to be passive participants in their own healthcare, complying with healthcare professional orders without question (Longtin et al. 2010). Today, there are calls for patients to be more active participants, who partner with healthcare professionals, evaluate the care provided, and join health research teams (Ocloo and Matthews 2016). This transformation has occurred through a series of pivotal moments in industry, culture, and technology. In this commentary, I explore how patients have come to shape hospital care at every level, from the bedside to the boardroom, and challenge readers to imagine how central their voice will be to the future of nursing and hospitals.

### 1.1 Defining Patient Participation

Patient participation, which is core to patient‐centred care, can be defined as delivering healthcare *with* patients, rather than merely *to* or *for* them (Ocloo and Matthews 2016). It emphasises a partnership between patients and healthcare professionals (Ocloo and Matthews 2016). It is important to acknowledge the diverse terminology used in this area with terms like patient, client, consumer, expert by experience, public and service user used synonymously, while terms for participation can include involvement, engagement and partnership (Longtin et al. 2010). The terms patient and public are used in this commentary, which is consistent with the Patient‐Centred Outcomes Research Institute (PCORI) in the United States and the Canadian Institutes of Health Research (CIHR) Strategy for Patient‐Oriented Research (SPOR), which are leaders in advocating for high levels of patient participation. However, I acknowledge that the term patient may align with a more passive and paternalistic model of care, which is a limitation of this term.

Patient participation can occur across multiple levels including the micro, meso, and macro levels of organisations (Castro et al. 2016). At the micro‐level, participation involves individual patients engaging directly in care activities with healthcare professionals at the bedside (Ocloo and Matthews 2016). The meso‐level focuses on interactions between patients or members of the public and the organisation (e.g., healthcare institutions, networks, or agencies), shaping the design, delivery, and evaluation of health services (Ocloo and Matthews 2016). Finally, macro‐level participation refers to collective involvement aimed at influencing broader healthcare systems (Ocloo and Matthews 2016). Here, patients and the public collaborate with national bodies or researchers to shape policies and practices that impact large populations.

## 2 Then: Selected Past Trends That Have Shaped Patient Participation

I will explore three societal trends that I have selected that have shaped patient participation in hospitals. However, I recognise that there are numerous reasons why patient participation has become more central at all levels of hospital care.

### 2.1 Demand for Quality

A key trend that has shaped patient participation in hospitals has been the evolution of quality management within healthcare. Released in 1999, the landmark report *To Err is Human* highlighted the risks and safety issues in hospitals by showcasing the 98,000 preventable deaths occurring in USA hospitals (de Jonge et al. 2011), which cemented patient safety as a healthcare quality movement. Leaders like Dr. Donald Berwick were instrumental in adapting quality improvement methods to healthcare organisations, like the Plan‐Do‐Study‐Act cycles, Lean and Six Sigma (de Jonge et al. 2011).

Interestingly, the evolution of quality management began in non‐healthcare industries and was borrowed by healthcare. An early example is the Japanese car industry. In the 70s and 80s, companies like Toyota were pioneers in quality control approaches, including lean manufacturing and continuous improvement cycles like the Plan‐Do‐Check‐Act cycle. A noteworthy initiative was Toyota's Dynamic Assurance System, developed in the 70s in response to public scrutiny that automobile companies were hiding defects and issues from the public. This system tracked defects in real‐time, provided this feedback to customers and the company, allowing for learning and improvement. While originating in manufacturing, healthcare soon recognised parallels, which resulted in cross‐learning, exemplified in hospital performance measurements and responses to failure, including feedback to patients.

Systems‐level quality improvement programs have become central to nursing practice. For example, in the early 2000s, the Transforming Care at the Bedside Initiative encouraged nurses to identify, test, and implement novel ideas to improve care, with one main focus area being enhancing patient participation. There was also the Releasing Time to Care: Productive Wards Initiative developed in the mid‐2000s and still in use at many hospitals today, which uses a modular improvement framework to increase the time patients spend with nurses, ultimately improving patient experience. Overall, these programs have paved the way for embedding patient participation into practice in response to gaps identified in care quality.

### 2.2 The Rise of Consumerism

Concurrent with the rise of the quality movement, consumerism, and the idea of thinking of patients as consumers have shaped patient participation practices in hospitals (de Jonge et al. 2011). The term consumer, which emerged from the private business sector, implies that patients have preferences, expectations, and experiences that must be met to ensure value, while also highlighting their right to information and choice. The rise of consumerism in healthcare mirrors what occurred in retail, hospitality, banking, and travel industries, where meeting consumer expectations became a central business strategy. As a result, consumers came to expect personalised, transparent services, with timely access and the ability to compare options. Patients began to expect the same from healthcare, putting pressure on these systems to adapt and be more responsive to patient needs.

Looking at key milestones, patient advocacy gained momentum in the 1980s, catalysed by the HIV/AIDS pandemic. During this period, patients challenged traditional hierarchies by demanding access to experimental therapies and participating in drug trial committees. This marked a pivotal moment: patients were leveraging their unique insights into unmet service needs to bring about change. Soon after, consumerism entered the health policy agenda, in part spurred by patient advocacy. For example, in the UK, conservative governments introduced internal markets and managerial oversight in the NHS to ensure responsiveness to patient feedback (Faculty of Public Health, 2010). Further, by the early 90s, a Patient Charter was developed for the NHS, which set explicit standards for service; like other industries, patients were now consumers with a service guarantee (Faculty of Public Health, 2010).

Understanding patient experience has become central to nursing practice and reflects a broader shift toward consumer‐oriented healthcare. For example, complaint departments in hospitals were pivotal in the 70s and 80s, focused on resolving disputes from those who were dissatisfied with their experience (Latimer et al. 2017). Around the same time, nursing scholars were leading the charge in qualitative research that aimed to understand patient experience using methodologies like phenomenology and grounded theory. Their seminal work was pivotal in shaping modern understanding of patient experience, as they showed that patient perceptions were critical to outcomes. By the 1990s, hospitals too were recognising the importance of patient feedback, collecting data from both those who were satisfied and those who were dissatisfied, as a tool to understand the service they were providing. A good example of this was the development of the HCAHPS survey in the United States, which measured standardised concepts of patient satisfaction and allowed benchmarking of care across hospitals and services. For the first time, patients' views could be compared systematically, creating accountability and a driver for quality improvement.

### 2.3 The Digital Revolution

It would be impossible to ignore the role of the internet in the growing trend of patient participation. Before the internet, patients relied heavily on nurses and other healthcare professionals for information, reinforcing traditional power dynamics. This began to shift in the early internet era (1990s–2000s), when early adopters started using a range of sources for health information, including both personal (friends and family) and impersonal sources (books, media, and the internet). However, with the rise of the digital age, by the mid‐2000s the internet quickly became mainstream and the most common and first source of health information, far surpassing other sources. Patients could look up symptoms, explore treatment options, and connect with others who shared their condition, even before talking with healthcare professionals. Overall, the internet has shifted information access from healthcare professionals to patients and enabled patients to be more confident participating in their healthcare.

The 2000s introduced new forms of digital engagement. Perhaps one of the most significant shifts in hospital care was the increasing patient access to personalised clinical data. Historically, clinical data were controlled by healthcare professionals, and patients were often discouraged from viewing their own records. This changed with the increasing adoption of electronic patient portals in the 2000s. These secure online platforms are tethered to hospital electronic medical records and allow patients to view their clinical data and identify errors in their care (Dendere et al. 2019). Another example is mobile apps, which were growing in popularity by 2010, providing patients with tools to enhance communication with healthcare professionals and self‐management practices. By 2015, wearables like smartwatches became accepted, providing patients with real‐time health insights, enabling patients to be more data‐informed. And in the 2020s, online symptom checkers became mainstream, especially in response to COVID‐19, shifting self‐care to the patients and minimising contact with healthcare professionals. COVID‐19 also led to a rise in QR code use in some countries, resulting in initiatives like access to question builders that helped patients plan questions when interacting with healthcare professionals (Tobiano et al. 2024). Continuing this momentum, the recent rise of artificial intelligence (AI) means patients can now ask AI questions about their health, helping them refine and prepare their concerns and engage more effectively during in‐person clinical encounters. Overall, the integration of digital tools has revolutionised how patients access information, facilitating more proactive and sophisticated patient participation.

## 3 Now and What's to Come? Examples of Patient Participation in Hospitals, Current Challenges and Future Considerations

These three broad trends have translated into current‐day patient participation at micro, meso, and macro levels of hospitals. While there are many examples of patient participation in current hospital care, I will focus on three examples below, discuss the current challenges, and propose where we might head in the future.

### 3.1 Speaking Up (Micro‐Level Participation)

#### 3.1.1 Example

Individual patients are shaping the care they receive by speaking up to influence communication at the point of care. By asking questions about their health or reporting concerns to a nurse, care can be altered. One example of this is patient participation in detecting clinical deterioration and escalating care. Patients or caregivers can use escalation systems to summon healthcare professionals to assess the patient's clinical condition promptly, often by calling a phone number linked to a healthcare professional not currently providing direct care, providing an avenue for raising safety concerns (Albutt et al. 2017). The implementation of bedside handover also encourages patients to speak up and is an example of a signature practice that has emerged from many system‐level nursing quality improvement programs mentioned previously. When nurses change shifts, instead of conducting the shift report in a private office away from the patient, nurses stand at the patient's bedside and engage the patient in a discussion about their care. This allows patients to be more informed, a passive approach to participation (Tobiano et al. 2018). However, bedside handover also facilitates more active participation; patients can identify and speak up about errors in the information being transferred, add information, and ask questions (Tobiano et al. 2018). Hospital initiatives like bedside handover have transformed the way patients access information, laying the foundation for patients to play a pivotal role in their own safety.

#### 3.1.2 Challenges

While many patients feel able to speak up to escalate care or take part in bedside handover, many barriers persist. Several individual patient factors influence this, such as patient age, health status/presence of chronic condition, familiarity with hospital and history of experiencing an adverse event. Further, the perceived lack of medical knowledge and trust in healthcare professionals also limits patient participation. Additionally, the nurse can impede patient participation. For example, in bedside handover, many nurses express discomfort with patient participation due to fears of increasing time, assumptions that patients may not be capable or may add irrelevant information, and confidentiality concerns (Tobiano et al. 2018). These fears can lead nurses to undertake approaches that actively discourage patient participation, such as standing in the doorway or corridor rather than at the bedside (Tobiano et al. 2018). Similarly, nurses may perceive escalation systems negatively, viewing them as undermining their professional judgement, reducing control, burdening patients and families, and leading to inappropriate or unnecessary calls that increase workload (Thiele et al. 2020).

#### 3.1.3 Future Considerations

Evidence consistently shows that nurses who actively facilitate patient participation are among the strongest influencers of higher levels of patient engagement. Therefore, it is essential to foster hospital cultures where nurses adopt a patient‐centred mindset. However, this is no easy feat. One of the leading indicators of patient‐centred care in hospitals is organisational leadership that genuinely supports and models this practice (Luxford et al. 2011). Ultimately, future efforts must focus on creating top‐down approaches to embed patient‐centred care across hospital systems if we are to see sustained patient engagement in safety behaviours like bedside handover and escalation systems.

### 3.2 Understanding Patient Experience (Meso‐Level Participation)

#### 3.2.1 Example

Today, patients share their experience of care often through qualitative‐type insights and quantitative measures. For the former, patients and the public often share their narratives and reflections through hospital committees and advisory bodies where they review safety and quality data and advise on protocols, materials, and service design. Quantitatively, Patient‐Reported Experience Measures (PREMs) capture what happened to patients in their interactions with the health system, offering an accurate reflection of the quality of care delivered. PREMs are now widely regarded as a robust measure, with strong evidence linking high patient experience to improved outcomes such as safety, adherence to treatment, and overall health (Bull et al. 2022). While these two types of feedback offer a direct link between patients/the public and system‐level decision‐making, for true patient participation to occur, organisations need to act on feedback received.

#### 3.2.2 Challenges

Currently, the depth of organisational action in response to patient feedback remains variable, which limits the extent to which patients can influence healthcare. Although patients and the public may have structural inclusion in hospital committees, research indicates that patients and the public often remain silent in their roles, with their contributions frequently overlooked or undervalued (Liang et al. 2018). This lack of recognition leads many to feel disconnected from genuine partnership in service improvement. Professional barriers play a critical role; of the 10 distinct obstacles identified in the literature, most stem from healthcare professionals themselves (Liang et al. 2018). These include negative attitudes toward patient/public involvement, limited knowledge and skills in engagement practices, and entrenched power dynamics within leadership teams (Liang et al. 2018). In terms of PREM data, challenges also persist, hindering meaningful use of PREM data. For example, if survey results are not provided to staff promptly, the usefulness of these data is questionable. Additionally, many organisational barriers hinder staff from analysing or acting on data, as competing priorities, limited resources, and insufficient management support leave little time for meaningful engagement in quality improvement initiatives.

#### 3.2.3 Future Considerations

Together, these insights highlight the need for both cultural and procedural shifts in the future. For hospital committees, transparent feedback loops, where committees report back to patients/public on how their input was used can enhance patient participation (Liang et al. 2018). More radically, advisory bodies and committees could be co‐led by patients, providing patients and the public with shared authority to sign off on decisions, reflecting a truly collaborative approach. For PREM data, the future will likely bring digital solutions that capture patient experience in real time using AI‐powered analytics (Bull and Pole 2024). Ideally, these insights could be displayed live on clinical units, enabling patients and nurses to interpret and act on them immediately. However, clinical areas must view the use of PREM data as core work to drive meaningful improvements in patient outcomes. Clinical leaders will require support to enact continuous quality improvement cycles in response to the real‐time data they receive (Bull et al. 2022). These quality improvement initiatives should be engaging and innovative to ensure PREM data is not viewed as punitive, but instead a powerful tool for improving care. To prepare for this, a culture of patient‐centred care in organisations is required (Bull et al. 2022), where nurses embrace the dimensions of patient experience, such as respect, communication, and patient participation, as this is what matters most to patients. Without this, experience data may be viewed as secondary to clinical outcome data.

### 3.3 Co‐Researching (Macro‐Level Participation)

#### 3.3.1 Example

Beyond service delivery, patients and the public are also influencing the very research that informs nursing practice in hospitals. Patients and the public can participate as team members in the research process, to improve the relevance of research for end‐users, and enhance the impact and translation of the research (Chudyk et al. 2022). This shift also brings significant benefits to the quality and rigour of the research process (Chudyk et al. 2022). Beginning in the UK and Canada in the early 2000s, people began to realise that research about patients must be conducted with patients. Around this time, the development of the James Lind Alliance was a key moment, where INVOLVE, the Royal Society of Medicine, and the James Lind Library, the James Lind Alliance developed methodology to bring patients and clinicians together to prioritise the most important uncertainties they faced related to care and treatments (Partridge and Scadding 2004). As more countries begin to adopt the mindset that all research must be conducted with patients, there is a shift away from the traditional model of academics working in isolation to decide what is research and how it is researched.

#### 3.3.2 Challenges

Despite growing recognition of the value of patient and public engagement in research, several challenges persist, such as power imbalances and time pressures. Patients and the public consistently express a desire for genuine engagement where their voices are heard and valued as true team members. However, building authentic relationships to facilitate this takes time, something often constrained by tight project timelines, limited funding, and lack of structural support. As a result, for nursing researchers, patient engagement is sometimes perceived as an added burden rather than an integrated part of the research process, especially when support is lacking. The importance of power is also critical, to ensure tokensim does not occur. Patients and the public emphasise that collaboration should go beyond symbolic gestures, advocating for mutual partnerships where hierarchical distinctions dissolve and their contributions are seen as valuable. With patient and public involvement becoming increasingly common and expected in research, nurse researchers are navigating changing times.

#### 3.3.3 Future Considerations

In terms of the future for nursing researchers, we are likely to overcome these barriers and progress rapidly up the IAP2 (International Association for Public Participation) Spectrum of Patient Participation, with traditional boundaries between patients and nurse researchers becoming increasingly blurred. Barriers to patient and public participation like power imbalances will be addressed in the near future, with patients and the public becoming comfortable and confident in research methods and jargon. For example, university‐led programs like PaCER at the University of Calgary provide patients and the public with research skills, such as qualitative data collection and analysis skills. As a result, patients and the public are successfully leading research projects, with nursing researchers playing a supportive role. As patients and the public increasingly take on leadership roles in research, nurses will need to adjust to these new ways of working.

## 4 Conclusions

Changes in quality improvement, consumer expectations and digital tools have played a part in how patient participation now occurs in hospitals. As a result, patients have moved from being silent to speaking up when concerned, from being studied to co‐leading research. If this momentum continues, the boundaries between patients' lived experience and professional expertise will likely become increasingly blurred. For nursing, this presents both a challenge and an opportunity: to embrace discomfort, relinquish control, and reimagine care, governance and research as co‐created endeavours. Overall, in 50 years, hospital care will not be built for patients, but by patients, and nurses must be ready to collaborate.

## Author Contributions

G.T.: conceptualization, data curation, writing – original draft, writing – review and editing.

## Funding

Georgia Tobiano was employed with funding from the NHMRC Centre for Research Excellence Grant APP1196436.

## Ethics Statement

The author has nothing to report.

## Consent

The author has nothing to report.

## Conflicts of Interest

The author declares no conflicts of interest.

## Data Availability

The author has nothing to report.
